# EXPLORING MEALTIME EXPERIENCES OF FAMILIES LIVING WITH DEMENTIA IN LONG-TERM CARE: A GROUNDED THEORY STUDY

**DOI:** 10.1093/geroni/igae098.2132

**Published:** 2024-12-31

**Authors:** Heather Alford, Allison Cammer, Heather Ward, Jason Perepelkin

**Affiliations:** University of Saskatchewan, Saskatoon, Saskatchewan, Canada; University of Saskatchewan, Saskatoon, Saskatchewan, Canada; University of Saskatchewan, Saskatoon, Saskatchewan, Canada; University of Saskatchewan, Saskatoon, Saskatchewan, Canada

## Abstract

Relationship-centered mealtime interventions improve staff-resident relationships and reduce the risk of malnutrition for residents living in long-term care settings. Less is known about the nature, goals, or outcomes of family-resident relationships at mealtimes, particularly for residents living with dementia. This constructivist grounded theory study aimed to explore what is needed to support meaningful family relationships and engagement at mealtimes with residents living with dementia in long-term care homes. Individual interviews were conducted with 13 family caregivers of people living with dementia in long-term care homes in a Western Canadian province. Interviews were recorded, transcribed verbatim, and analyzed using constant-comparison method. Three themes characterized family member participants’ experiences of mealtimes: 1) Changing Ways of Connecting, 2) Nutrition as a Reflection of Dementia Progression, and 3) Adjusting Expectations. Mealtimes are important opportunities for supporting family-resident connection and can facilitate ‘acceptance’ of the illness trajectory in dementia among caregivers. By conceptualizing their mealtime involvement, this study advances understanding of the essentiality of family caregivers for enhancing relationship-centered practices in long-term care settings. By explicating the structure, function, and experience of family involvement in nutritional care, this study addresses the current gap in understanding of how families living with dementia build and preserve relationships through nutritional care and dining.

